# Acute Stressors Reduce Neural Inhibition to Food Cues and Increase Eating Among Binge Eating Disorder Symptomatic Women

**DOI:** 10.3389/fnbeh.2016.00188

**Published:** 2016-10-13

**Authors:** Zhenyong Lyu, Todd Jackson

**Affiliations:** ^1^Key Laboratory of Cognition and Personality, Southwest UniversityChongqing, China; ^2^Department of Psychology, University of MacauMacau, China

**Keywords:** binge eating, fMRI, acute stress, external food cues, food consumption, cognitive control

## Abstract

Stressors can trigger binge-eating but researchers have yet to consider their effects on both neural responses to food cues and food consumption among those at risk. In this experiment, we examined the impact of acute stressors on neural activation to food images and subsequent food consumption within binge-eating disorder (BED) and non-eating disordered control groups. Eighteen women meeting DSM-IV BED criteria and 26 women serving as non-eating disordered controls were randomly assigned to unpleasant stressor (painful cold pressor test (CPT) followed by negative performance feedback) or less unpleasant stressor (non-painful sensory discrimination task followed by positive performance feedback) conditions. Subsequently, they were scanned with functional magnetic resonance imaging (fMRI) while viewing food and neutral images. After the scans, participants completed a self-report battery in an environment conducive to snacking. During exposure to food images, BED-symptomatic women in the unpleasant stressor condition reported more liking of high calorie food images and showed less activation in one inhibitory area, the hippocampus, compared to controls in this condition. BED-symptomatic women exposed to unpleasant stressors also consumed more chocolate than any other group during the post-scan questionnaire completion. Crucially, reduced hippocampal activation to high calorie food images predicted more chocolate consumption following fMRI scans within the entire sample. This experiment provides initial evidence suggesting unpleasant acute stressors contribute to reduced inhibitory region responsiveness in relation to external food cues and later food consumption among BED-symptomatic women.

## Introduction

Theory and research have linked unpleasant stressors to binge-eating episodes within binge-eating disorder (BED) symptomatic groups yet associated neural responses are not well understood. One plausible hypothesis is that stressors enhance reward region responsiveness to external food cues, precipitating increases in consumptive behavior. Alternately, unpleasant stressors might decrease activation in regions associated with inhibition or cognitive control in the presence of such cues, ultimately fostering increased food consumption. Towards clarifying this issue, we assessed the impact of unpleasant acute stressors on neural responses to external food images and food consumption in average weight BED-symptomatic women and non-eating disordered controls.

BED is characterized by episodes of consuming unusually large amounts of food accompanied by a perceived loss of control. Affected persons experience marked distress related to bingeing but do not engage in compensatory behaviors such as purging, fasting, or excessive exercise following binges (American Psychiatric Association, 2013). Lifetime prevalence in the USA was estimated at about 3.5% for women and 2.0% for men (Hudson et al., 2007). However, binge-eating disturbances have also become increasingly common in highly populated non-Western nations such as China (Chen and Jackson, 2008; Jackson and Chen, 2010; Tong et al., 2014). For example, Tong et al. (2014) estimated 3.53% of young Chinese women met criteria for BED in a two-stage epidemiological study of eating disorder prevalence in Wuhan China. BED is distinct from other eating disorders and obesity, but psychiatric and medical comorbidity is common (Grilo et al., 2009).

The affect regulation model offers one explanation of links between stressors and BED symptoms (Hawkins and Clement, 1984; Haedt-Matt and Keel, 2011; Leehr et al., 2015). From this perspective, elevations in negative affect trigger binge eating episodes because food provides comfort and distraction from distress. Furthermore, given that binge eating is effective in reducing negative affect, at least in the short-run, future risk for binge-eating is increased (Haedt-Matt and Keel, 2011). As such, negative affect is especially likely to induce binge-eating among people who have binged previously compared to non-binge eaters. Support for the model hinges, in part, upon data demonstrating relatively high or rising levels of unpleasant affect prior to binge episodes among binge-eaters.

Presumably, unpleasant stressors are a common source of negative affect among binge-eaters. Related research has implicated acute stressors as influences on increased food consumption, particularly of high-fat and high-sugar foods (Epel et al., 2001; Dallman et al., 2003, 2005); for example, elevations in perceived stress, reported hassles and stressful life events predict more frequent binge eating episodes and increases in unhealthy eating (Wolff et al., 2000; Crowther et al., 2001; Pendleton et al., 2001; O’Connor et al., 2008; Klatzkin et al., 2015; Mason and Lewis, 2015; Zhu et al., 2016). Stress has also been implicated both in increasing vulnerability to BED maintenance (Striegel-Moore et al., 2002).

Illustrating possible causal effects of stress, Gluck et al. (2004) found that an obese BED group reported more hunger and a stronger desire to binge eat than obese non-BED controls did following a cold pressor test (CPT). Laessle and Schulz (2009) assessed effects of a threatening social stressor, the Trier Social Stress Test vs. a neutral task (reading a newspaper for the same duration) on the subsequent rate and duration of pudding consumption in BED patients and non-BED controls. The BED group ate more rapidly and consumed more pudding though, notably, this difference was isolated to a more stressful condition (Laessle and Schulz, 2009). Later, Schulz and Laessle (2012) reported a BED-symptomatic group showed more motivation to eat and less deceleration of eating in a stressful compared to a less stressful condition while non-BED controls had a complementary pattern. Together, these studies suggest that BED-symptomatic groups are especially prone to over-eating following exposure to unpleasant stressors.

Regardless, neural responses underlying stressor-consumption relations are not well understood. One plausible hypothesis is that stressors differentially affect reward circuitry responsiveness to external food cues among BED-symptomatic groups compared to non-disordered controls. Reviews of neuroimaging literatures on obesity and eating disturbances have implicated the medial orbitofrontal cortex (OFC), caudate, putamen, nucleus accumbens (NAc), ventral striatum and insula as key reward areas with increased activation interpreted as evidence for enhanced reward region responsiveness to external food cues and/or food anticipation (Rolls, 2004; Schäfer et al., 2010; Carnell et al., 2012; Alonso-Alonso et al., 2015). Results can vary on the basis of reward region operationalizations, hunger state and task (e.g., passive viewing vs. imagined eating; Martin et al., 2010; Dimitropoulos et al., 2012; Frankort et al., 2012) but these contentions have drawn support from functional magnetic resonance imaging (fMRI) research wherein an overweight BED group reported more reward sensitivity and showed stronger medial OFC responses while viewing food images compared to a bulimia nervosa (BN) group, obese controls, or average weight controls (Schienle et al., 2009). Subsequently, using positron emission tomography, Wang et al. (2011) found obese BED patients had more dopaminergic activity than did controls in the caudate/putamen during exposure to food stimulation.

Alternately, acute stressors and increases in unpleasant affect might result in reduced responsiveness of brain regions implicated in cognitive control or behavior inhibition among BED-symptomatic persons exposed to external food cues. General reviews of response inhibition studies (Gray and McNaughton, 2000; Buchsbaum et al., 2005; Simmonds et al., 2008) and those specific to obesity and eating disturbances (Benoit et al., 2010; Carnell et al., 2012) have identified the dorsolateral prefrontal cortex (DLPFC), superior frontal gyrus (SFG), middle frontal gyrus (MFG), inferior frontal gyrus (IFG), ventrolateral prefrontal cortex (vlPFC), hippocampus, and/or anterior cingulate cortex (ACC) as potentially important inhibitory control regions.

Representative research from Yokum and Stice (2013) found cognitive reappraisal strategies aimed at suppressing appetitive responses to palatable food images increased inhibitory region activity in the SFG, DLPFC and vlPFC. Marsh et al. (2011) concluded that diminished IFG and ACC activity contribute to reduced control over eating among BN patients. Finally, aside from its hypothesized involvement in general behavior inhibition (Gray and McNaughton, 2000), some researchers have linked impaired hippocampus functioning to heightened food intake, increased appetitive behavior and problems inhibiting responses to external food cues (e.g., Tracy et al., 2001; DelParigi et al., 2004; Davidson et al., 2009).

Despite evidence implicating food reward and cognitive control regions in binge-eating responses, it is not known whether acute stressors contributing to unpleasant affect increase reward region activation and/or reduce activity in cognitive control areas in the presence of external food cues nor is it clear which activation differences between BED-symptomatic groups and controls are salient to subsequent food consumption levels. Moreover, despite evidence suggesting that substantial percentages of people with BED are not obese (Kessler et al., 2013), very little is known about behavioral and neural responses within non-obese BED-symptomatic groups due to the near exclusive reliance upon obese BED-symptomatic groups within neuroimaging research.

Highlighting links between obesity and BED symptoms, select USA-based questionnaire research has estimated nearly 70% of those who report binge eating also endorse a body mass index (BMI) in the obese range (Grucza et al., 2007), yet rigorous, large-scale multinational research based on structured interviews has reported substantially lower obesity rates within subgroups fulfilling a DSM-IV BED diagnosis. Specifically, Kessler et al. (2013) assessed more than 24,000 participants from 14 mostly upper middle and high income Western countries including the USA They found 63.7% and 58.3% of those who met all criteria for BED during their lifetimes and past 12 months, respectively, were not obese. A substantial minority (26.5%) diagnosed with BED within the past 12 months even had a BMI in the average range. While the point prevalence information on BMI at time of diagnosis was not reported, these data suggest BED is not inevitably related to being obese and substantial percentages with the diagnosis are not obese.

On a related note, cross-national obesity data underscores how USA obesity prevalence estimates do not reflect obesity rates in highly populated non-Western nations including China. Flegal et al. (2012) reported an age-adjusted mean BMI of 28.7 for USA men and women aged 20 years and older in 2009–2010; more than one third of those sampled (35.7%) were obese. In contrast, the 2011 China Health and Nutrition Survey (Mi et al., 2015) reported an age-adjusted mean BMI of 23.8 for mainland Chinese adult men and women in the same age range and a far lower age-adjusted obesity rate (11.3%). Other recent China-based epidemiological research concluded that eating disorder rates among young Chinese women are similar to those found in Western nations (Tong et al., 2014) while mean BMIs of adolescent and young adult Chinese subgroups with binge-eating pathology have more typically fallen at the low end of the average range not the obese range (e.g., Chen and Jackson, 2008; Jackson and Chen, 2010, 2015). Based on these data, the continued neglect of non-obese BED-symptomatic samples within neuroimaging research seems unfounded, particularly when considering large Asian nations where much of the planet’s population is concentrated.

Based on the preceding review, we examined the impact of acute stressors on: (1) neural activation responses to visual food cues as well as; (2) post-fMRI food consumption levels of average weight BED-symptomatic groups and non-eating disordered controls. Drawing upon assumptions of the affect regulation model and previous research (Laessle and Schulz, 2009; Schulz and Laessle, 2012), we hypothesized that BED-symptomatic participants exposed to unpleasant stressors would eat more chocolate subsequently than BED-symptomatic participants exposed to neutral stressors or non-eating disordered controls in either of these conditions. Second, within the unpleasant stressor condition, we expected BED-symptomatic participants would show more activation than controls in a priori-selected reward/motivation regions of interest (ROIs; i.e., OFC, putamen, caudate, vmPFC, nucleas accumbens and/or insula) and/or less activity in ROIs reflecting response inhibition (i.e., DLPFC, SFG, MFG, IFG, hippocampus, ACC) during exposure to visual food images. Conversely, in the neutral stressor condition, fewer salient ROI activation differences were expected between the BED-symptomatic group and controls. Finally, we hypothesized that ROIs differentiating BED-symptomatic groups from controls during fMRI scans would predict later chocolate consumption levels within the entire sample.

## Materials and Methods

### Participants

The final sample included 18 women who endorsed all DSM-IV BED criteria on a validated eating disorder screen (Stice et al., 2000) and 26 women who endorsed few eating disorder syndrome criteria on this screen served as non-eating disordered controls. Data of two other women who participated (one each from BED-symptomatic and control groups) were excluded due to motion artifacts during their scans (>2.5 mm). Furthermore, we elected to exclude data from two men who also met all BED criteria from the main analyses, given the sharp gender disparity. Respondents ranged in age between 18 and 23 years (*M* = 19.65 years, *SD* = 1.27) and were predominantly of Han majority ethnicity (82.6%). The mean BMI of respondents was 19.80 (*SD* = 1.97, Range: 16.16–24.14). None of the BED-symptomatic women had a BMI lower than 17.5 (range: 17.62–24.14). Exclusion criteria included metallic implants, claustrophobia, current psychopharmacological medication, current or past psychiatric diagnosis aside from BED and presence of a current medical condition. All participants were right-handed non-smokers with normal or corrected-to-normal vision. No group differences in menstrual cycle phase were found. Written informed consent was obtained from each participant before entry into the study, which was approved by the human research ethics committee of Southwest University, China.

### Stimuli

Digital color images depicting high-caloric foods (e.g., French fries, ice cream, cake, chips), low-caloric foods (e.g., cucumbers, carrots), and neutral images (i.e., cars) were used in this study. Each category included 90 different images. Complexity, brightness, and color composition were matched among the three categories based on recent related work (Jackson et al., 2014).

### Questionnaire Measures

#### Eating Disorder Diagnostic Scale (EDDS; Stice et al., 2000)

This 22-item self-report scale was based on DSM-IV criteria for Anorexia Nervosa, BN and BED and was used to identify participants endorsing all BED criteria as well as ruling out a BED diagnosis among those in the non-disordered control group. The scale has excellent reliability, a high level of stability over 2 weeks, and excellent concordance with diagnoses based on structured interviews and self-report measures of disordered eating (Stice et al., 2000, 2004).

The EDDS has also been used extensively in identifying eating disorder-symptomatic adolescents and young adults in large mainland Chinese samples (Jackson and Chen, 2007, 2010; Chen and Jackson, 2008) and has high positive correlations with weight-based body image disturbances and eating disorder risk factors in Chinese adolescents and young adults of each gender (Jackson and Chen, 2008, 2010, 2011, 2014, 2015). The full EDDS was used as a screen to identify BED-symptomatic and non-disordered control subgroups. Participants also completed the five EDDS items assessing binge-eating criteria during the formal experiment to confirm the status as BED-symptomatic or control group members. The alpha coefficient for these five items was *α* = 0.75 in the final sample.

#### Uncontrolled Eating Scale (UES; Karlsson et al., 2000)

The nine-item UES of the Three-Factor Eating Questionnaire-R18 was included as an additional continuous measure of binge eating to evaluate the distinctiveness of BED-symptomatic women vs. non-disordered controls. Items were rated on a four-point likert scale and summed to derive total scores. The measure has sound reliability and validity in past studies (e.g., Karlsson et al., 2000; de Lauzon et al., 2004). Its alpha was *α* = 0.88 in this sample.

#### Image Pleasantness

After the fMRI scans, participants rated each image on a 9-point scale assessing pleasantness (1 = not at all, 9 = very much). For each image type (high calorie food, low calorie foods, cars) ratings were summed into total scores.

#### Demographics

Age, gender, ethnicity (Han majority vs. ethnic minority), menstrual cycle phase and objective measures of height and weight were assessed.

### Behavior Measures

The amount of a popular, name brand chocolate consumed by each participant during the post-fMRI assessment was measured on the basis of total weight in grams (g).

### Procedure

The day before their scan, participants were instructed to consume regular meals, but refrain from drinking caffeinated beverages for 12 h and to avoid eating for at least 3 h before the experiment. BED-symptomatic women and controls were randomly assigned to unpleasant and less unpleasant stressor control conditions. Women in the unpleasant stressor condition completed two CPT trials. Specifically, they were asked to immerse their non-writing hand in ice water maintained at 3.5°C for as long as possible but to remove the hand when it became too uncomfortable. There was a 4 min time limit for each trial with a 3 min break between trials. The CPT has been widely used as a stress test (Kelly and Cooper, 1998) and produces stronger cortisol responses in BED groups (Gluck et al., 2014). BED-symptomatic women and controls did not differ in hand immersion durations averaged across the two CPT trials, *t* = −0.31, *p* = 0.97 (*M* = 37.00 s, *SD* = 40.30 vs. *M* = 37.58 s, *SD* = 44.75). Because negative performance feedback is also widely used in stress-induction paradigms, and reliably induces negative affect and anxiety (Stroud et al., 2002; Bogdan and Pizzagalli, 2006), after each trial, all women in this condition were also told that they had done poorly compared to most others who previously performed the task.

Women in the less unpleasant stressor condition were asked to complete two “temperature detection” task trials (2 min immersions in room temperature water with a 3 min break between trials). To better ensure the task was perceived as less unpleasant, all participants in this condition were also given positive performance feedback indicating that they performed better than most other people who had previously completed the task.

As a manipulation check after each task, women in each stressor condition rated how: (1) stressful; (2) painful; and (3) unpleasant the task was on 11-point (0–10) scales with *0 = Not at all* and *10 = Very much so* as anchors. Supporting the integrity of the experimental manipulations, women in the unpleasant stressor condition rated their task to be more stressful (*M* = 4.61, *SD* = 2.04 vs. *M* = 2.70, *SD* = 2.11), *F*_(1,44)_ = 9.73, *p* = 0.003, painful (*M* = 6.98, *SD* = 2.21 vs. *M* = 1.13, *SD* = 1.45), *F*_(1,44)_ = 112.47, *p* < 0.001, and unpleasant (*M* = 5.52, *SD* = 1.97 vs. *M* = 3.91, *SD* = 2.36), *F*_(1,44)_ = 6.31, *p* = 0.016, than did their peers in the control condition.

Following stress inductions, fMRI scans were undertaken. Each scan comprised two runs, including three blocks of each image type (high calorie food, low calorie food, cars), respectively. Each block included 15 images presented for 2 s with an inter-stimulus interval (ISI) of 0.5 s. Before and after each block, a white fixation cross was presented in the middle of the screen for 16 s. Participants were told to simply watch every image on the screen.

After their scans, participants were taken to a waiting room to have their height/weight assessed and complete the measures described above. All self-report measures had been back-translated previously for use in Chinese samples (Jackson et al., 2014). The waiting room was standardized to include bowls of brand-name milk chocolate and bottled water. To enhance this environment as one conducive to food consumption, standardized baskets with empty chocolate wrappers and water bottles, presumably from previous participants, were on display. After handing a participant the post-task research measures, the first author stated, “If you’d like, you can help yourself to snacks and water while you’re finishing these”, and left her alone for 20 min. After participants completed the experiment and left, amounts of chocolate consumed were assessed and newly empty chocolate wrappers were replaced before the arrival of the next participant.

Prior to debriefing, the women were asked to guess the main research purpose; none of them identified binge eating, different stressor conditions, or amount of chocolate consumed as the foci of the experiment. Debriefing followed and featured a description of the general research purposes and time to answer participant queries. Typically, the experiment took 50 min to complete. Sixty yuan was paid as compensation.

### fMRI Data Acquisition

Scans were performed with a Siemens TIM Trio 3T MRI system equipped with a standard 12 channel head coil (Siemens Magnetom Trio TIM, Erlangen, Germany). An echo-planar imaging (EPI) sequence was used with 432 T2*-weighted images recorded per run (*TR* = 2000 ms; *TE* = 30 ms; flip angle = 90°; FOV = 192 × 192 mm^2^; matrix size = 64 × 64; voxel size = 3 × 3 × 3 mm^3^; interslice skip = 0.99 mm; Slices = 32). T1-weighted images were acquired with a total of 176 slices at a thickness of 1 mm and in-plane resolution of 0.98 × 0.98 mm^2^ (*TR* = 1900 ms; *TE* = 2.52 ms; flip angle = 9°; FOV = 250 mm^2^ × 250 mm^2^).

### Design and Data Analysis

#### Demographic and Behavioral Measures

All analyses were performed using SPSS Version 20. Group differences on demographics (age, ethnicity, education, BMI) were assessed via chi-square analysis or one-way analysis of variance (ANOVA). Significant demographics were to be included as covariates in subsequent behavior and fMRI analyses. One way ANOVAs assessed group differences on continuous measures of bingeing behavior, uncontrolled eating, image pleasantness and post-fMRI chocolate consumption, controlling for any group differences on demographics. Bonferroni-adjusted *post hoc* tests assessed specific group differences when *F*^′^s were significant.

#### fMRI Data

fMRI data were analyzed in the context of General Linear Modeling (GLM) on a voxel by voxel basis via SPM8 (Friston et al., 1994) in MATLAB (Mathworks, Inc., Sherborn, MA, USA; Worsley and Friston, 1995). Data were normalized to the Montreal- Neurological-Institute template in 3 mm^3^ × 3 mm^3^ × 3 mm^3^ voxel sizes, and smoothed with a 6-mm kernel full-width-at-half-maximum. Image types (high-calorie foods, low calorie foods, cars) were modeled by a function convolved with a hemodynamic response function (HRF) in the GLM. Six movement parameters applied by the realignment procedure were introduced as covariates in the first-level GLM. The time course of brain activation was modeled with a boxcar function convolved with the canonical HRF and a temporal derivative function. A first order autoregressive model was also implemented to correct for autocorrelations in error terms of the fMRI model.

Following associated research (Schienle et al., 2009), a two-stage analysis procedure was used within a mixed-effects design. At the first level, fMRI data from each woman generated statistical contrasts for comparing brain activation to: (1) High-calorie food vs. car (HiCal-Car) images; (2) Low-calorie food vs. car (LoCal-Car) images; and (3) high-calorie food vs. low-calorie food (HiCal-LoCal) images. These contrasts were then entered into second level analyses to compare each BED-symptomatic group with controls within the same stressor condition. Contrasts between conditions of interest were assessed with *t* statistics.

Pre-specified ROIs noted above were based on past reviews (Born et al., 2010; Bruce et al., 2010; Martin et al., 2010). Those related to reward sensitivity/motivation and food reward included the OFC, putamen, caudate, vmPFC, nucleas accumbens, amygdala and insula while ROIs reflecting behavioral inhibition and cognitive control were the DLPFC, SFG, MFG, IFG, vlPFC, hippocampus and ACC. ROI masks were generated using the AAL-atlas (Tzourio-Mazoyer et al., 2002) as implemented in the WFU-pickatlas toolbox (Maldjian et al., 2003). Corrected *p* values were reported for exploratory analyses (*p* < 0.05, false discovery rate corrected, FDR) while uncorrected* p* values (*p* < 0.001) were reported for ROI effects based on associated recent peer-reviewed studies (Stice et al., 2010; van der Laan et al., 2014; Coveleskie et al., 2015; García-García et al., 2015). The minimum cluster size threshold was set to* k* = 5, also following related peer-reviewed work (Schienle et al., 2009; Jovanovic et al., 2013; Jackson et al., 2014). Finally, within the whole sample, we assessed associations between ROIs that significantly differentiated groups in response to visual food cues and post-fMRI chocolate consumption levels.

## Results

### Behavioral Data

No group differences were found on ethnicity (*χ*^2^ = 4.17, *p* > 0.05) or age, education and BMI (Table 1). One-way ANOVAs indicated each BED-symptomatic subgroup reported more binge-eating behavior and uncontrolled eating than control subgroups did, supporting the distinctiveness of these subgroups. Only one group difference was found *vis a vis* image pleasantness ratings: control group women in the unpleasant stressor condition rated high-calorie food images to be less pleasant than peers in any of the other conditions (*p* < 0.05). Finally, for post-fMRI chocolate consumption, BED-symptomatic women in the unpleasant stressor condition ate significantly more chocolate than women in each of non-disordered control group did (*p*’s < 0.05), and marginally more chocolate than BED-symptomatic women in the control condition (*p* = 0.051).

**Table 1:** **Group differences on demographics, self-report measures and chocolate consumption**.

	BED-HI (*n* = 9)	BED-LO (*n* = 9)	CON-HI (*n* = 12)	CON-LO (*n* = 14)	*F*	Group differences
Age (years)	19.22 (0.44)	19.89 (1.54)	20.00 (1.41)	19.43 (1.34)	0.87	no differences
Education (years)	13.00 (0.00)	13.33 (0.71)	13.17 (0.58)	13.21 (0.58)	0.58	no differences
BMI	20.80 (1.48)	20.72 (2.34)	19.19 (1.52)	19.22 (2.16)	2.34	no differences
Binge behavior	3.78 (0.97)	3.11 (1.05)	1.25 (1.06)	0.64 (0.63)	28.31***	BED-HI > CON-HI, CON-LO***; BED-LO > CON-HI, CON-LO***
UES	24.00 (3.04)	23.44 (4.67)	17.00 (4.08)	18.43 (4.60)	6.46***	BED-HI > CON-HI, CON-LO**; BED-LO > CON-HI, CON-LO**
High-calorie food pleasantness	6.26 (0.82)	6.58 (1.28)	4.49 (1.33)	6.39 (1.17)	8.34***	BED-HI > CON-HI**; BED-LO > CON-HI***; CON-LO > CON-HI***
Low-calorie food pleasantness	5.01 (1.74)	4.86 (1.93)	4.96 (1.19)	5.63 (0.77)	0.51	no differences
Car pleasantness	4.99 (1.87)	4.58 (1.61)	4.70 (0.79)	5.36 (1.46)	0.56	no differences
Chocolate consumed (grams)	43.56 (37.98)	21.78(21.13)	15.17 (17.36)	7.00 (14.27)	4.85***	BED-HI > CON-HI, CON-LO**; BED-HI > BED-LO^†^

Note: BED-HI, binge eating disorder symptomatic group in unpleasant stressor condition; BED-LO, binge eating disorder symptomatic group in less unpleasant stressor condition; CON-HI, non-eating disordered control group in unpleasant stressor condition; CON-LO, non-eating disordered control group in less unpleasant stressor condition; BMI, body mass index; UES, uncontrolled eating scale; ^†^p < 0.10; **p < 0.01; ***p < 0.001. All p-values were based on two-tailed significance tests.

### fMRI Data

#### Activation Differences in the Unpleasant Stressor Condition

**Figure 1:**
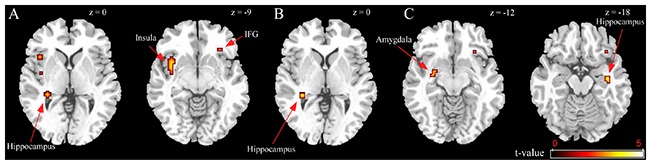
**Group differences in unpleasant stressor condition. (A)** High-caloric food vs. car contrast; **(B)** Low-caloric food vs. car contrast; **(C)** High-caloric food vs. Low-caloric food contrast.

BED-symptomatic women in the unpleasant stressor condition showed significantly less IFG, insula and hippocampus activation than controls did during exposure to high calorie food images relative to low calorie food or car images (Table 2). The former group also experienced less hippocampus activity in the low calorie food vs. car contrast. Finally, BED-symptomatic women showed comparatively less hippocampus, IFG and amygdale activation in the HiCal-LoCal contrast condition (see Table 2, Figure 1).

**Table 2:** **Regions of interest (ROI) reaching significance (*p* < 0.001) in Group × Stressor condition analyses**.

Condition contrast and region	BA	Hem	Cluster Size	*x*	*y*	*z*	*t*
**Unpleasant stressor**
**CON > BED**
**High-caloric food vs. car**
Inferior Frontal Gyrus	47	R	6	30	27	−9	3.00
Insula	13	L	56	−33	6	−9	3.68
Hippocampus	−	L	9	−30	−36	0	3.44
**Low-caloric food vs. car**
Hippocampus	−	L	13	−30	−36	0	4.22
**High- vs. Low-caloric food**
Hippocampus	−	R	13	39	−15	−18	4.12
Inferior Frontal Gyrus	−	R	5	27	24	−9	3.63
Amygdala	−	L	11	−27	−6	−12	3.79
**Less unpleasant stressor**	
**CON > BED**
**High-caloric food vs. car**
ACC	32	L	10	−3	36	6	3.58
Superior Frontal Gyrus	6	R	18	24	9	48	3.93
**Low-caloric food vs. car**
Putamen	−	R	13	21	3	−3	3.75
ACC	−	R	7	15	39	12	3.42
Superior Frontal Gyrus	6	R	16	21	12	45	3.55
**High- vs. Low-caloric food**
Parahippocampal Gyrus	−	L	7	−21	−30	−21	3.88

*Note: BED, binge eating disorder symptomatic; CON, non-eating disorder control; IFG, Inferior Frontal Gyrus; ACC, anterior cingulate cortex*.

#### Activation Differences in the Less Unpleasant Stressor Condition

BED-symptomatic women in the less unpleasant stressor condition showed less ACC and SFG activation than controls did in HiCal-Car and LoCal-Car contrasts. BED-symptomatic women also had less parahippocampal gyrus activity in the HiCal-LoCal contrast. Contrary to predictions, the former group also showed comparatively weaker rather than stronger activity in select hypothesized reward ROIs (left OFC, putamen) for HiCal-Car and LoCal-Car contrasts, respectively (see Table 2).

#### Correlations Between ROIs and Chocolate Consumption

**Figure 2:**
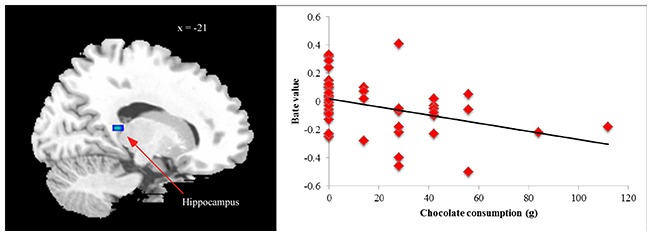
**Correlation between chocolate consumption and hippocampus activation [MNI coordinates *x*, *y*, *z*: −21, −39, 9] in high- vs. low-calorie food image contrast**.

Finally, we examined relations between ROIs that differentiated BED subgroups from controls in between-groups analyses and post-fMRI amounts of chocolate eaten within entire sample. No outliers (3 SDs ± mean) were found for total chocolate consumed. No significant ROIs from HiCal-Car or LoCal-Car contrasts were related to chocolate consumption (all *p*’s > 0.05). However, supporting its role as an inhibitory control region, less hippocampus activation in the HiCal- LoCal contrast condition predicted more subsequent chocolate consumed in the sample [MNI coordinates *x*, *y*, *z*: −21, −39, 9; *r* = −0.37, *p* = 0.012] (see Figure 2). Even after dichotomizing chocolate consumption subgroups (no chocolate eaten vs. chocolate eaten), reduced hippocampus activity was related to eating rather than not eating chocolate, *t* = −2.46, *p* = 0.012.

## Discussion

Exposure to acute stressors and increases in unpleasant affect can precipitate binge-eating episodes among BED-symptomatic persons, but neural responses underlying related experiences have never been examined within this literature. Towards illuminating this issue, we assessed the extent to which reward area activity increases and decreased inhibitory region responsiveness to external food cues contributed to this pattern in BED-symptomatic women vs. non-eating disordered controls.

Regarding links between BED symptomatology, unpleasant stressors and post-fMRI chocolate consumption levels, results supported hypothesized group differences. As expected, BED-symptomatic women and controls randomly assigned to the unpleasant stressor condition experienced more pain, stress, and unpleasant affect in response to associated tasks than did peers in the less unpleasant stressor control condition. Supporting tenets of the affect regulation perspective (Haedt-Matt and Keel, 2011; Leehr et al., 2015), BED-symptomatic women exposed to unpleasant stressors also ate more chocolate following their scans than did non-disordered controls or complementary groups in the control stressor condition. In fact, the former group consumed more than twice as much chocolate, on average, than did BED-symptomatic women in the control condition (*p* = 0.051) and about 300–600% more chocolate than non-eating disordered subgroups did. While researchers have linked stress and daily hassles to binge-eating (Crowther et al., 2001; O’Connor et al., 2008), the pattern observed here underscored how exposure to unpleasant stressors triggers more eating in women who report previous binge-eating than non-binge eaters or binge-eaters not exposed to such triggers (Hawkins and Clement, 1984). Reassuringly, Laessle and Schulz (2009) previously observed this pattern in obese samples but the current findings indicate this facet of the affect regulation model extends to average weight BED-symptomatic women as well. Taken together, these studies suggest that reported history of binge-eating rather than weight status or the synergy of binge-eating and obesity may be the more critical influence on overeating following unpleasant stressors in BED-symptomatic samples.

Hypothesized ROI activation differences reflecting response inhibition also received support. Specifically, within the unpleasant stressor condition, BED-symptomatic women experienced less activation than controls did in one inhibitory ROI, the hippocampus, across all three image contrast conditions. Critically, reduced hippocampus activation during exposure to high calorie images also predicted significantly more post-fMRI chocolate consumption within the entire sample. Past work has found neurohormonal signals related to meal initiation (e.g., ghrelin), meal cessation (e.g., cholecystokinin), and feedback on the status of energy stores (e.g., leptin, insulin) all have receptors in the hippocampus (Lathe, 2001); these appear to modulate hippocampal-dependent learning and memory processes (Zhao et al., 2004; Diano et al., 2006; Harvey et al., 2006). Aside from its possible role in appetitive processes, the hippocampus is involved in regulating neuroendocrine stress responses (Pruessner et al., 2008; Ulrich-Lai and Herman, 2009). For example, fMRI evidence from Pruessner et al. (2008) indicated hippocampus activity was curtailed in response to a stressor, in turn, contributing to hypothalamic-pituitary-adrenal axis initiation and stress hormone release.

Animal researchers have found rats with hippocampal lesions have general learning deficits that require suppression of previously learned responses (Winocur, 1980) in addition to increased food intake, weight gain, appetitive behavior, metabolic activity, and difficulty inhibiting responses to external food cues when compared to intact controls (Tracy et al., 2001; Davidson et al., 2009). On this basis, Davidson et al. (2009) argue that impaired hippocampal activity underlying such deficits interferes with the ability to use conditional cues evoked by food or food-related environmental cues and interoceptive satiety signals in suppressing appetitive and consumption responses. Hippocampus damage in humans can also interfere with the capacity to use intero- and extroceptive cues in satiety signaling (Born et al., 2010). One fMRI study found obese and formerly obese people had lower hippocampal blood flow than did lean controls after consuming a liquid meal to satiation (DelParigi et al., 2004). Extending such work, the present results underscored how reduced hippocampus responsiveness to *external* food cues following an unpleasant stressor differentiated a non-obese BED-symptomatic group from non-obese controls and helped to explain the higher post-fMRI chocolate consumption level of the former group.

In contrast to this difference in inhibitory region responsiveness, there was no evidence that *increased* responsiveness in reward/motivation ROIs discriminated BED-symptomatic women from controls exposed to unpleasant stressors. In fact, the former group showed significantly less activation during exposure to high calorie food images in the putamen, an area implicated in elevated reward motivation as well as the insula which has links with taste (Carnell et al., 2012). Explaining the functional significance of these structures on this basis would suggest exposure to the more unpleasant tasks corresponded to less activation of reward/taste regions in response to high calorie food images among BED-symptomatic participants.

However, such an interpretation seems problematic because pleasantness ratings for high calorie food images and chocolate consumption levels were elevated among BED-symptomatic participants compared to controls in the unpleasant stressor condition. The putamen and insula have multiple functions, aside from those reflecting reward and taste. Consistent with behavior results and the interpretation of hippocampus findings, the putamen has also been identified as a key inhibitory region in separate meta-analyses of structures involved in response inhibition during go/no-go tasks (Buchsbaum et al., 2005; Simmonds et al., 2008). In addition, insula activity differences were not localized to the anterior region associated with taste but rather the left posterior insula (BA13); increased activity in this area corresponds to the anticipation and experience of negative affect (Richieri et al., 2011; Aupperle et al., 2012) in addition to pain processing (Rainville, 2002). In this light, reduced BA13 responsiveness among BED-symptomatic women exposed to unpleasant stressors might have reflected comparatively less aversive affect evoked by high calorie food rather than neutral images.

In the less unpleasant control condition, the hypothesis that BED-symptomatic and non-disordered groups would have fewer ROI activation differences was not supported. Instead, BED-symptomatic women in this condition also showed relatively lower activation to food images in hypothesized ROIs reflecting cognitive control (i.e., the SFG, ACC, parahippocampal gyrus), albeit none of these predicted later chocolate consumption levels in the sample. Comparatively reduced putamen activity in this group mirrored results for the other BED-symptomatic subgroup, though the effect emerged for low- rather than high calorie images. Also paralleling results for the unpleasant stressor condition, BED-symptomatic women exposed to the less unpleasant stressors did not show enhanced reward ROI responsiveness to food images. Rather, this subgroup had lower ACC activity than did controls in the high-calorie food-car contrast. Reduced anterior cingulate metabolism has been linked to impaired inhibitory control and risk for overeating among healthy obese persons (Volkow et al., 2009), but this study may be the first to implicate these areas in average BMI persons with binge-eating concerns. Taken together, the present results and those of Volkow et al. (2009) suggest implications of these activation differences should be explored in future longitudinal work on risk for later weight gain and obesity.

Notwithstanding possible implications of this study, several limitations should be noted as foundations for extensions. First, while the sample *N* was more than twice as large as those used in 26 of 29 fMRI studies included in a review of responses to visual food cues (van der Laan et al., 2011), within cell n’s were quite modest; therefore extensions of the current paradigm to larger samples with more power may aid in detecting subtle neural responses. Second, even though results for BED-symptomatic women were based on endorsing all DSM-IV BED criteria from a validated diagnostic screen, BED diagnosis can be established only from structured interviews with follow-up probes. Eating disorder assessment via anonymous surveys can increase candor (Keel et al., 2002) and the EDDS has excellent concordance with diagnostic interviews (e.g., Stice et al., 2000) but interview-based assessment should be considered in extensions to determine how well findings apply to women with a definitive BED diagnosis.

Third, standardized procedures and random assignment were employed to control for potential stressor condition differences in background functioning. However, the inclusion of pre-experiment hunger ratings would have been preferable in assessing and ruling out group differences in hunger as an influence on results. Fourth, in relation to external validity, a considerable subset of women with BED and binge-eating tendencies may not be obese (Jackson and Chen, 2010; Kessler et al., 2013; Tong et al., 2014) and assessment of average weight samples here underscored links of reported BED symptoms with hypothesized behavior and neural activation responses independent of obesity. However, obesity is also common in BED and BED-symptomatic samples (Grucza et al., 2007; Kessler et al., 2013). Furthermore, findings may not generalize to predominantly male samples, given evidence of gender can affect ability to inhibit brain activation elicited by food stimuli (Wang et al., 2011). Extensions to obese groups, men, and samples from non-Asian contexts can elucidate relative contributions of neural responses associated with reward and cognitive control more fully. Finally, possible causal links between acute stressors, neural responses to food images, and subsequent chocolate consumption were examined on a single occasion lasting less than 1 h. As a result, inferences about long-term consequences of such associations are necessarily tentative and warrant consideration in extensions based on more costly prospective designs.

## Conclusion

In sum, this experiment supported key assumptions of the affect regulation perspective of binge-eating. In line with tenets of the model, BED-symptomatic women exposed to unpleasant stressors subsequently consumed more chocolate following exposure to external food images relative to non-eating disordered controls or BED-symptomatic women exposed to less unpleasant stressors. Imaging results for the unpleasant stressor condition indicated reduced activation to food cues in one inhibitory ROI, the hippocampus, differentiated the BED-symptomatic group from controls and predicted higher chocolate consumption levels in the entire sample. While BED-symptomatic women in the less unpleasant control stressor condition also showed reduced inhibition ROI activity relative to controls, there was no evidence that enhanced reward ROI responsiveness differentiated BED-symptomatic groups from controls in either stressor condition or predicted post-fMRI chocolate consumption. As such, this study provides initial evidence suggesting reduced hippocampal responsiveness to external food cues helps to explain why exposure to unpleasant stressors magnifies vulnerability to later overeating among the BED-symptomatic.

## Author Contributions

TJ designed the project. ZL performed the experiment and analyzed the data. ZL and TJ wrote the manuscript.

## Conflict of Interest Statement

The authors declare that the research was conducted in the absence of any commercial or financial relationships that could be construed as a potential conflict of interest.
